# Therapeutic potential of exosomes derived from mesenchymal stem cells for treatment of systemic lupus erythematosus

**DOI:** 10.1186/s12950-024-00381-2

**Published:** 2024-06-12

**Authors:** Shima Famil Samavati, Reza Yarani, Sara Kiani, Zohreh HoseinKhani, Masomeh Mehrabi, Steven Levitte, Rosita Primavera, Shashank Chetty, Avnesh S. Thakor, Kamran Mansouri

**Affiliations:** 1 Medical Biology Research Center, Kermanshah University of Medical Sciences, Kermanshah, Iran; 2 Translational Type 1 Diabetes Research, Department of Clinical Research, Steno Diabetes Center Copenhagen, Gentofte, Denmark; 3 Interventional Regenerative Medicine and Imaging Laboratory, Department of Radiology, Stanford University School of Medicine, Palo Alto, CA 94304 USA

## Abstract

Autoimmune diseases are caused by an imbalance in the immune system, producing autoantibodies that cause inflammation leading to tissue damage and organ dysfunction. Systemic Lupus Erythematosus (SLE) is one of the most common autoimmune diseases and a major contributor to patient morbidity and mortality. Although many drugs manage the disease, curative therapy remains elusive, and current treatment regimens have substantial side effects. Recently, the therapeutic potential of exosomes has been extensively studied, and novel evidence has been demonstrated. A direct relationship between exosome contents and their ability to regulate the immune system, inflammation, and angiogenesis. The unique properties of extracellular vesicles, such as biomolecule transportation, biodegradability, and stability, make exosomes a promising treatment candidate for autoimmune diseases, particularly SLE. This review summarizes the structural features of exosomes, the isolation/purification/quantification method, their origin, effect, immune regulation, a critical consideration for selecting an appropriate source, and their therapeutic mechanisms in SLE.

## Introduction

Autoimmune diseases (ADs) are among the most significant chronic diseases leading to morbidity and mortality in patients worldwide [1, 2]. Although the exact drivers of ADs are unknown, genetic factors, infections, and environmental factors are considered triggers or exacerbators. The frequency of ADs patients is 90% in women [3] and are classified into two types: organ-specific, where a single organ is affected, such as type 1 diabetes mellitus (T1DM) [4], multiple sclerosis (MS), and psoriasis [5]; and systemic, in which the immune response simultaneously affects different organs and tissues, such as systemic lupus erythematosus (SLE), Sjögren’s syndrome (SS), and rheumatoid arthritis (RA).

SLE is a clinically heterogeneous autoimmune disease that affects multiple organs, including skin, joints, heart, kidneys, central nervous, and hematologic systems, causing irreversible organ damage. The prevalence of SLE varies from 20 to 150 cases per 100,000 individuals, with a higher incidence in women (9:1) [6]. In SLE, abnormal immune cell activation leads to organ damage [7, 8]. Following the loss of self-tolerance to autoantigens, autoreactive T cells, and B cells promote autoantibodies’ production and immune complex deposition throughout the body, leading to end-organ dysfunction [9]. In this multifactorial disease, genetic/ epigenetic/ hormone and environmental factors interact to modulate the clinical phenotypes. Some susceptibility loci have been detected, including complement components C1q and C4, major histocompatibility complex (MHC) (especially the human leucocyte antigen, class II), T cell receptor, and many cytokines (IL-6, IL-27, IL-12, IL-23) [10, 11]. Interestingly, some SLE susceptibility loci are shared with other autoimmune diseases such as diabetes and rheumatoid arthritis, such as PTPN22 (protein tyrosine phosphatase 22) and STAT4 (signal transducer and activator of transcription 4), which may explain why SLE patients are at higher risk of developing other autoimmune conditions [12]. Epigenetic dysregulations found in many SLE seem to play a role both in disease initiation and progression, including altered pathways of DNA methylation pattern [13, 14], histone acetylation [15], and microRNAs [16, 17]. Moreover, a strong association has been detected between SLE and environmental factors such as pesticides, Epstein-bar virus, endometriosis, and even postmenopausal hormone therapy, which may trigger autoimmune responses and modulate alternating periods of disease flares in SLE [18–20].

Although there has been tremendous progress in drug treatment strategies for controlling SLE, the current treatment approach still relies centrally upon nonsteroidal anti-inflammatory drugs (NSAIDs), glucocorticoids (GCs), and immunosuppressive agents. Unfortunately, these common drugs only moderately increase survival, and their use is associated with poor outcomes and frequent recurrence, which may reflect the inadequacy of disease-modifying therapeutic approaches. SLE is known for its relapsing and remitting courses, with a life-long costly burden for patients [21]. Finally, the side effects of current treatments can be severe: NSAIDs, for example, cause serious side effects, including gastrointestinal bleeding from ulcers and myocardial infarction. Managing these side effects on top of the underlying disease presents a unique challenge for patients and their physicians [6, 22, 23].

As another relatively recent auxiliary/ alternative treatment option, cell-based therapies, such as stem cell transplantation, are gaining interest in treating severe diseases. However, stem cell transplant remains a last resort due to the high rate of serious adverse events and extraordinary financial cost, highlighting the need for safer and more effective therapies [24, 25].

Exosomes are abundantly and ubiquitously found in biological bodies in physiological fluids performing essential roles in transmitting intermediate cellular messages. They have recently emerged as a promising therapeutic approach for immunotherapy with additional promise in regenerative medicine [26]. Exosomes are now recognized to significantly activate, suppress, and surveil immune pathways [27]. They serve as valuable immune responses in various diseases such as cancer, cardiovascular disease, nephropathy, and autoimmune diseases. Accordingly, we briefly describe biological characteristics, biogenesis, isolation, detection, functional activities, advantages and disadvantages of exosomes, and how this promising new therapy could be transformative in treating SLE.

## Exosomes characteristics

Extracellular vesicles (EVs) include different types of vesicles that are classified based on their size, morphology, biogenesis (exosomes, microvesicles, and apoptotic bodies), flotation density, chemical composition, and the presence of marker proteins such as Alix, TSG101, flotillin 1, HSP70, and CD9 [28, 29] (Table 1).

**Table 1:** Biological features of extracellular vesicles

Type	Exosomes	Microvesicles or Ectosomes or Prostasomes	Apoptotic bodies
Diameter (nm)	40–130	100–1000	50–5000
Origin	Multivesicular bodies	Cell membrane	Apoptosis Programmed death
Release	Endocytosis	Budding	Budding
Receptors	Tetraspanins (CD63, CD9, CD81), Alix, TSG101, Annexins, flotillin, Hsp60, Hsp70, Hsp90	Integrins, selectins, metalloproteinases, CD40 Phosphatidyl-serine	Phosphatidyl-serine
Cargos	miRNA, miRNA, lncRNA, circRNA, mtDNA, lipid raft MHC molecules metabolize enzymes ribosomal protein etc.	mRNA, miRNA, lipid raft membrane receptors, cytoplasmic proteins (cytokines),	Nuclear fractions, cell organelles, DNA, rRNA, mRNA
Sedimentation	100,000-130,000 ⨉ g	16,000–25,000 ⨉ g	5,000–16,000 ⨉ g

Hsp: heat shock proteins; MHC: major histocompatibility complex; mRNA: messenger RNA; miRNA: microRNA; rRNA: ribosomal RNA; TSG101: tumor susceptibility gene 101

Microvesicles, also known as ectosomes and prostasomes, range in size from 100 to 1000 nm and are released by direct germination (fission) of the plasma membrane [30]. Large structures called apoptotic bodies, up to 5000 nm, are released from the plasma membrane during apoptosis via direct budding. Exosomes are “nano biovesicles” (average size: 40–180 nm) released into surrounding body fluids upon fusion of multivesicular bodies and the plasma membrane. Specifically, they are derived from the internal budding of endosomes as they accumulate in intraluminal vesicles (ILVs), known as multivesicular bodies (MVBs). As the smallest EVs, exosomes possess unique characteristics over other nanocarriers, including immunomodulatory effects, biodegradability, longer circulatory half-life, and permeability across the blood-brain barrier (BBB) [27, 31, 32]. Furthermore, The exosome membrane is enriched in transmembrane markers CD9, CD63, CD81, and TSG101, RAB family proteins, all involved in vesicle trafficking and signal transduction [30], and cholesterol, sphingomyelin, phosphatidylserine, and glycosphingolipids [33].

Exosome entry to target cells occurs through receptor binding, direct membrane integration, and endocytic internalization [28]. These vesicles carry nucleic acids, including RNA, mitochondrial DNA, single- or double-stranded DNA, proteins, and lipids [33]. In the following section, we will discuss the methods of isolation and quantification of exosomes.

## Exosome purification and quantification

The first step of exosome functional analysis is ensuring purity. To date, many methods have been designed and developed for the purification of exosomes, including differential centrifugation, density gradient centrifugation, size exclusion chromatography (SEC), filtration, polymer-based precipitation, and immune affinity capture, microfluidic technologies; several commercially available exosome isolation kits have been developed [34]. Each approach has its advantages and disadvantages that directly correspond to exosome origin.

One of the most common exosome isolation methods is tandem and high-speed centrifugation and ultracentrifugation, which separate and purify the exosomes from large debris and dead cells, usually from culture media supernatant. This method is unsuitable for sources such as urine or serum due to the possibility of co-precipitation of aggregated protein and non-specific large binding proteins.

Centrifugation is typically followed by filtration using 100 nm filters to overcome contamination with small proteins. Although this could be regarded as a relative high-efficiency method, the contamination risks due to the fragmentation of microparticles into smaller vesicles due to filtration pressure are still high [35, 36]. Immunoaffinity methods bypass the protein contamination risk by using antibody-containing magnetic cell beads to enrich exosomes, but the yield is often low [37]. Therefore, ultracentrifugation is still considered the optimal method of purification and enrichment for exosomes from culture media, followed by a further purification step of enrichment through concentration gradients. However, these methods are time-consuming, labor-intensive, and require expensive equipment to achieve acceptable purity.

Recently, efficient and reproducible exosome isolation kits have been created, including ExoQuick (System Bioscience) [37], the Total Exosome Isolation kit (Invitrogen) [38], and Exospin (Cell Guidance System) [39], which operate based on particle size and reduce the purification time to less than 2 h.

After isolation, exosomes must be quantified and characterized, raising another challenge. Methods have been developed for determining the size, density, morphology, and composition of exosomes, including fluorescence-based detection, nanoparticle tracking analysis (NTA), resistive pulse sensing, dynamic light scattering (DLS), atomic force microscopy (AFM), transmission and scanning electron microscopy (TEM, SEM) [40–42].

The most common antibody-based techniques of exosome biophysical characterization include western blotting and enzyme-linked immunosorbent assay (Elisa). These methods detect intra-vesicular or membrane-bound protein markers, while real-time quantitative polymerase chain reaction (RT-qPCR) detects exosome-specific RNA molecules.

Although there are many advanced technologies for rapid isolation, purification, quality control, and identification of exosomes, there is still a need for more studies and validation of these methods, particularly regarding clinical applications.

## Exosomes origins and function

The activity and function of exosomes have been extensively studied. They are released by multiple cell types, including stem cells, T and B lymphocytes, macrophages, dendritic cells, neurons, endothelial cells, adipocytes, and epithelial cells under normal and pathological conditions [43]. Exosomes could be detected in all body fluids, including blood, semen, breast milk, ascites, saliva, lymphatic fluid, cerebrospinal fluid, and amniotic fluid at concentrations between 1.13 and 1.19 g/ml [44, 45]. Depending on their origin and cargo, they are endocyted by the target cells, where they initiate downstream effects [46]. The most prominent effects exerted by exosomes can be observed in the integration of neurons and glial cells in the central nervous system (CNS) [47]; coagulation, angiogenesis, and thrombosis in the cardiovascular system; regulation of antigen presentation [48], activation of T-cells and polarization to regulatory T cells, immune suppression, and anti-inflammatory systems in the innate and acquired immune system [49]. Additionally, immunosuppressive cells of both myeloid and lymphocyte origins (regulatory T cells) could be induced by released exosomes from mesenchymal stromal cells (MSCs) [50].

Other than being involved in the regulation of immune-related pathologies [51], analysis of exosome’s coding and non-coding nucleic acids (mRNAs, miRNAs, non-coding RNAs, tRNAs, rRNAs, mitochondrial DNA, single- or double-stranded DNA) have confirmed a close relationship between exosome distribution and cell differentiation, cell survival, and repair [51–53].

Previously, Tan and colleagues found that exosomes released by antigen-presenting cells (APCs), such as B cells, DCs, and macrophages, boost antigen-presenting function. On the other hand, exosomes released from T cells induce immunoregulatory effects [54, 55]. As cell-free therapeutic particles, exosomes demonstrate lower immunogenicity due to diminished transmembrane protein expression, specifically MHC [56]. Since exosomes do not replicate, they cannot contribute to chromosomal abnormalities, genetic transformation, or tumor formation.

This, in turn, established the exosomes as a prime candidate for use in the treatment of systemic diseases such as systemic lupus erythematosus (SLE) [57], multiple sclerosis (MS) [58], diabetes [4], and Sjögren’s syndrome (SS) [59]. Such immunomodulatory effects, specifically from a mesenchymal stem cell (MSC)-derived exosomes, are exerted by directing macrophages and T cells into M-2 and Treg/ T_H_2 cells, respectively [60].

Their ability to pass biological barriers, affecting organs with physiological barriers in the brain and kidney, has also given them an edge for clinical applications over standard cell-based approaches such as MSC therapy [61].

The characteristic of exosomes, which are biocompatible and enter the cell quickly, compared to liposomes, which are less biocompatible and accumulate on the surface of some cell lines, such as HEK293, and enter the cell to a lesser extent, makes EV a suitable option to transfer drugs in all kinds of diseases [62].

In this way, engineered exosomes loaded with specific therapeutic molecules will increase the effectiveness of targeted therapy especially compared to liposomes. For example, engineered exosomes conjugated to curcumin [63] and cRGD-Exo [64] injected intravenously will markedly suppress the inflammatory response and cell apoptosis in ischemic brain lesions [65]. Thus, exosomes can be used therapeutically as vectors of various nucleic acids such as siRNAs and miRNAs, proteins, or even low molecular weight drugs. Encapsulated anticancer drugs in exosomes, such as doxorubicin and paclitaxel, have been used to treat brain tumors while minimizing systemic toxicity [66].

Exosomes have been shown to trigger the inflammatory response through pattern-associated molecular patterns (PAMP) receptors such as toll-like receptors (TLRs). TLRs are a group of receptors in the mammalian innate immune system that identify pathogenic invaders by adopting the best immune response [67]. TLRs 1, 2, 4, 5, and 6 are expressed on the cell surface, while TLRs 3, 7, 8, and 9 are placed in intracellular endosomes [68, 69]. Exosomes boost the regulatory functions of T cells [70], inhibit activation of natural killer cells (NK)/CD8 + T cells [71, 72], and promote differentiation/maturation of DCs [73]. A more recent application of exosomes clearly shows that exosomes from MSCs are potent in subsiding cytokine storm in severe Covid-19 patients, which is an intensified response of immune cells on both levels of activation and pro-inflammatory cytokine production [74]. In the next segment, we will describe the exosome’s structure, contents, formation, and interaction patterns by the target cell.

## Exosome composition and uptake

**Fig. 1:**
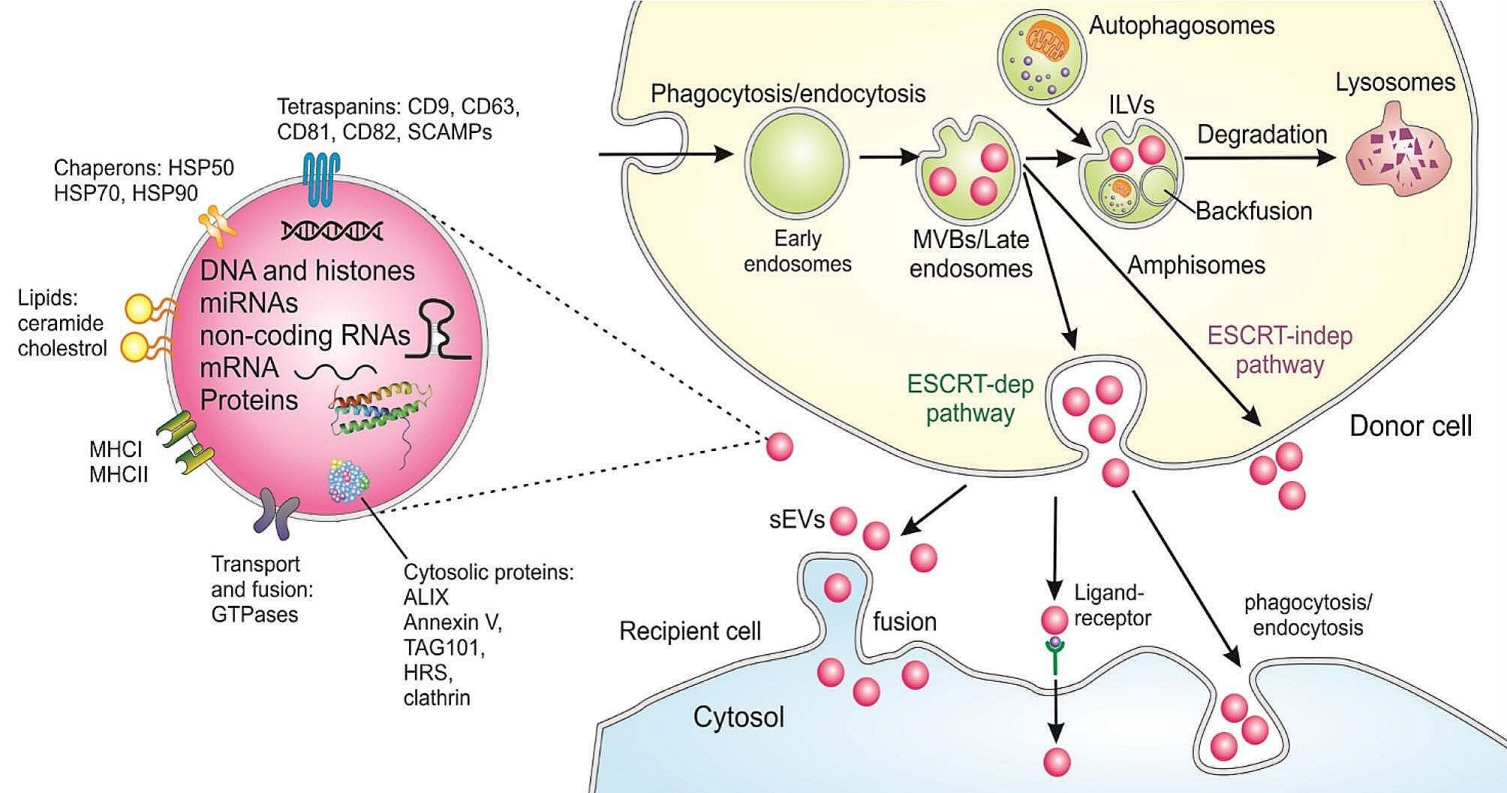
EV biogenesis, cargo contents, and uptake. HSP, heat shock proteins; SCAMPs, secretory carrier membrane proteins; Alix, Apoptosis-linked gene 2–interacting protein X; HRS, hepatocyte growth factor-regulated tyrosine kinase substrate; TSG101, tumor susceptibility gene 101

Exosomes function in various biological and pathological processes depending on their cargo. These nanostructures usually carry a range of biomolecules such as nucleic acids (RNAs, mitochondrial DNA, single- or double-stranded DNA), proteins, and lipids [75, 76]. As shown in Fig. 1, proteins detected in exosomes perform various functions, from transportation and membrane fusion (e.g., GTPases, annexins, and flotillins) to the biogenesis of multivesicular bodies (e.g., Alix, TSG101, and clathrin). Others could include tetraspanins (e.g., CD9, CD63, CD81, and CD82) [77, 78], heat shock proteins (e.g., Hsp70 and Hsp90), integrins, and RAB proteins. The latter protein family mediates the connection and integration of exosomes with target cells [79]. Moreover, lipids such as cholesterol, ceramides, prostaglandins, sphingolipids, and phosphatidylserines are asymmetrically distributed between the outer and inner EV membranes, although the functional ramifications of this remain unclear. The biogenesis of EVs is a complex process in which MVBs/late endosomes are formed in the endocytic pathway. During the inward budding of MVBs, many intraluminal vesicles (ILVs), specific proteins from the MVB membrane, and spheroids containing cytosolic compounds are formed [80]. The endosomal sorting complex required for transport (ESCRT) is employed to organize such a complex transport system of protein cargoes. This network comprises four subsets of ESCRT-0, -I, -II, and -III coordinated with one another via complementary proteins such as VPS4, VTA1, Alix, and TSG101 to accelerate the formation of MVBs and ILVs.

Exosomes can influence target cells in three ways: receptor binding, direct membrane integration, and endocytic internalization [28]. First, surface connection and transmission can occur without the need for cargo transfer and in an indirect manner, as detected during immune responses [81]. The second and direct way involves connection and integration with the membrane of the target cells to transfer mRNAs, miRNAs, proteins, and signaling molecules. Thirdly, exosome surface ligand interaction with the target cell receptors leads to their activation and downstream signal transduction by membrane proteins, surface adhesive molecules, and receptors, but without invagination [82]. The following part will describe the immunomodulatory effects of mesenchymal stem cell-derived exosomes.

## MSC-exosomes immunomodulatory effects

Mesenchymal stem cells (MSCs) are mature pluripotent cells that can differentiate into mesoderm tissues. Although these cells were initially isolated from the bone marrow (BM-MSCs), they can be isolated from other sources such as adipose tissue, dental pulp, cord blood [83], placenta, and so on [84]. The high proliferative potential and immunosuppressive properties of the MSCs have made them excellent clinical candidates for treating various conditions. MSCs’ ability to repair tissue is attributed to their released biological compounds in exosomes rather than direct cell replication/differentiation upon engraftment [85, 86]. Moreover, MSC-derived exosomes (MSC-Exos) have been found quite effective in the process of regeneration, specifically in the case of myocardial infarction, acute kidney damage, liver fibrosis, and neurological diseases such as Alzheimer’s [87] and amyotrophic lateral sclerosis (ALS) [88] both in vivo and in vitro [89–91]. MSC-Exo immunosuppressive properties of are exerted through the carried RNA and proteins, immunologically active agents such as anti-inflammatory cytokines, and regulating Toll-like receptor signaling (TLR) [92, 93]. Although MSC transplantation has been frequently suggested and studied in mouse models of autoimmune diseases such as SLE, studies are devoted to mesenchymal cell secretions or their purified exosomes for such purposes [94]. In a model of multiple sclerosis, MSC-Exos inhibited the proliferation of autoreactive lymphocytes and stimulated the secretion of anti-inflammatory cytokines, namely IL-10 and TGF-β [95]. Another therapeutic application of MSC-derived Exos is the amelioration of cutaneous and mucosal manifestations in therapy-refractory graft-versus-host disease (GVHD) by decreasing steroid use [96, 97].

Moreover, APC-released exosomes have also been considered applicable for therapeutic purposes [98]. Dendritic cell-derived Exos (DC-Exos) could also function as immunosuppressants when exposed to immunosuppressive drugs or cytokines. For example, DCs treated with IL-10 and IL-4 have been shown to reduce inflammation in collagen-induced arthritis via their exosome signaling [99, 100]. It has been stated that DC-derived exosomes could be as effective in treating arthritis and other autoimmune disorders as stem cell-derived exosomes [101, 102].

Altogether, exosomes now interest researchers considerably, irrespective of origin but rather based on their functions as delivery vehicles of nanoparticles. They are emerging as promising tools for their immune-regulating properties in treating autoimmune diseases [103, 104].

## Exosomes as a signal of SLE activity

**Fig. 2:**
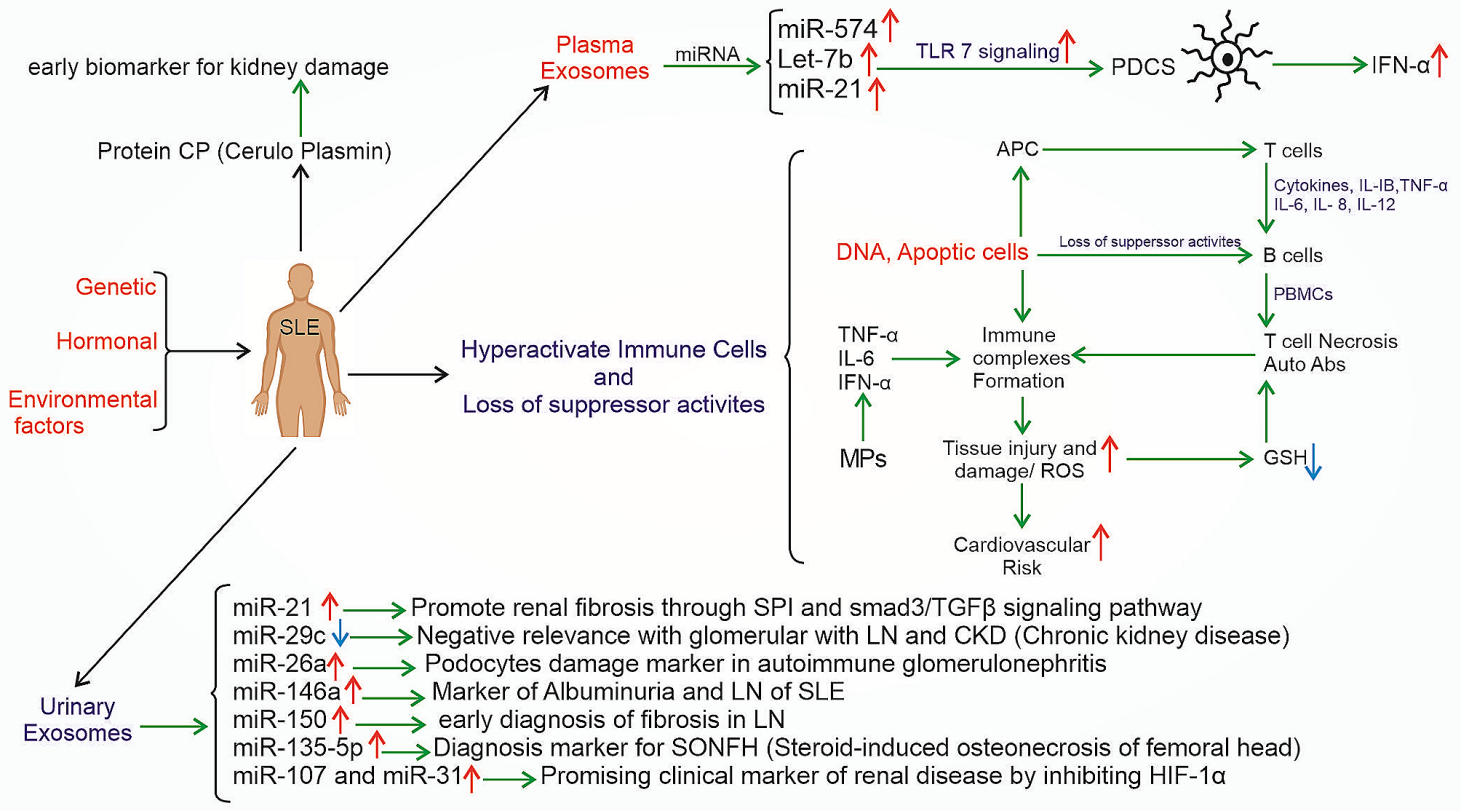
The development of systemic lupus erythematosus (SLE) and lupus nephritis (LN). The schematic diagram illustrates how hyperactivated immune cells cause disease progression, and serum and urinary EV components and miRNAs level could be regarded as disease initiation and progression markers. genetic and epigenetic factors trigger SLE formation. during disease progression, DNAs and apoptotic cells stimulate the activity of B cells by stimulating peripheral blood mononuclear cells (PBMCs) to produce TNF-α, IL-1β, and IL-6, which causes T cell necrosis and autoantibodies production. Moreover, T cell stimulates cytokine production (TNF-α, IL-1β, IL-8, IL- 12, and IL-6) through antigen-presenting cells (APCs) and B cell activation. microparticles (MPs) also increase IL-6, TNF-α, INF-α, and ultimately, autoantibodies production and tissue injury. High reactive oxygen species (ROS) production and reduced glutathione (GSH) also evaluated T cell necrosis and increased cardiovascular disease risk. In plasma exosomes, miRNAs (miR-574, let-7b, and miR-21) expression increases IFN-α production through TLR7 signaling via stimulating plasmacytoid DCs (pDCs). urinary exosomes, including miR-26a, miR-21, miR-29 C, miR-135b-5p, miR-107, miR-31, miR-146a, miR-150 promote renal fibrosis are candidate biomarkers for LN and SLE. additionally, cerulo plasmin (CP), a protein from urinary exosomes, could be regarded as an early biomarker to diagnose kidney disease

Higher immunoglobulin and complement system component concentrations have been found in SLE patients compared to healthy individuals [69, 70]. Accordingly, it could be concluded that the immune system in SLE patients releases “SLE-specific” endogenous nanocarrier exosomes that stimulate an immune response. Such extracellular vesicles are potentially valuable biomarkers of disease activity and severity [105]. T cell-derived exosomes, which are consist of some molecules such as miRNAs, long noncoding RNAs (lncRNAs), circular RNAs (circRNAs), and proteins made it as a unique biomarker for SLE progression. As depicted in Fig. 2, subpopulations of platelet, endothelial, and leukocyte-derived circulating exosomes of SLE patients activate plasmacytoid DCs (pDCs) with an expression of miR-574, let-7b, and miR-21 via TLR7 signaling, leave a specific signature that could be applied diagnostically and prognostically [69, 106, 107]. Moreover, exosomes detected in SLE patients exhibit decreased mitochondrial and platelet membrane proteins and increased glycolytic and cytoskeletal proteins that induce strong proinflammatory responses [108]. Studies have shown a strong association between SLE severity and increased plasma level of MPs (100 nm- 1 μm) [109]. The increased plasma level of MPs in SLE patients stimulates the release of proinflammatory cytokines (IL-6, TNF-α, and INF-α) from some dendritic cell subsets while increasing the number of CD 14 + monocytes. Interestingly, exosomes from healthy cases and patients with other autoimmune diseases have different characteristics [110, 111]. As circulating exosomes are immunologically active could stimulate TNF-α, IL-1β, and IL-6 production and convert B cells to T cell necrosis and autoantibodies through healthy peripheral blood mononuclear cells (PBMCs) in lupus [57]. Additionally, Perl et al. proved the abnormal mitochondria function in T lymphocytes of SLE patients with high reactive oxygen species (ROS) production and reduction in glutathione (GSH) eventuated T cell necrosis [112].

As mentioned earlier, IC deposition is a primary and critical event in the glomerulus of lupus nephritis (LN) patients with kidney damage. In 10–30% of SLE patients, it leads to organ failure and death [113]. Studies confirm the association between platelet-derived MPs in the formation of IC by harboring IgG, which contributes to disease activity pathogenesis [114]. In a study by Nielsen et al., the relationship between circulating MPs carrying high concentrations of galectin-3-binding protein (G3BP, lupus nephritis urinary marker) and disease activity was shown [115]. They suggested that targeting MPs’ surface molecules, such as G3BP, could alleviate inflammation and prevent IC formation by reducing extracellular autoantigens [116].

Studies have shown that urinary exosome microRNAs as cell-free biomarkers, that collected noninvasively could be used to diagnose kidney and genital diseases [117, 118]. In this regard, the amounts of exosomal miRNAs, such as miR-26a and miR-29c, in the urine of LN patients were detected to be increased and decreased, respectively, compared to healthy individuals. This change will increase urinary protein and renal fibrosis, reflecting podocyte and kidney damage [119, 120]. The presence of high levels of miR-21 can lead to the Programmed Cell Death 4 gene and regulate T cell activity in SLE patients. Moreover, it takes human plasmacytoid dendritic cells to IFN type І production [121].

Fortunately, researchers have designed an exosomal urinary multimarker panel that includes miR-150, miR-135b-5p, miR-107, miR-31 and miR-146a are used for early detection and prognosis of LN [122, 123]. For instance direct relationship between the miR-146a and chronic inflammatory parameters such as proteinuria and C3 and C4 complements activation have proved. It could negativelymodulate inflammation through TRAF6 and IRAK1 inhibition [124, 125].

In summary, the quantity and phenotype of circulating miRNAs exosomes could serve as potential biomarkers, predict the development and progress, and offer novel therapeutic approaches to SLE. In the next section, we will focus on exosome immunomodulatory/immunostimulatory mechanisms in patients with SLE.

## Signaling pathways of exosomes in SLE

The immunomodulatory effects of exosomes on innate and adaptive immune systems through subduing T cells, B cells, and macrophages, as well as boosting tissue regeneration, have prompted consideration as a novel therapeutic candidate for autoimmune inflammatory pathologies such as SLE [126, 127]. Fortunately, exosomes are less immunogenic than their cell source as they express lower numbers of immunogenic transmembrane proteins, such as MHC complexes [56].

One of the serologic hallmarks of SLE is the production of autoantibodies against nuclear molecules (anti-nuclear antibodies (ANAs)) [127, 128]. ANAs form circulating proinflammatory immune complexes (ICs), which trigger innate immune cells’ production of inflammatory cytokines. IC deposition in tissues (especially the kidneys) activates the complement system, stimulating inflammation and tissue damage [129]. Numerous reports of circulating IC-carrying microparticles (MPs) in SLE patients display DNA and nucleosomal molecules in an antigenic form [130]. Exosomes are considered an inhibitor against autoantibody production in SLE. On the other hand, exosomes have also been identified as proinflammatory mediators in SLE. Exosomes isolated from SLE patients have been found to trigger the peripheral blood mononuclear cells (PBMCs) from healthy individuals to produce TNF-α, IL-1β, and IL-6, all proinflammatory cytokines [57]. Dieker et al. showed in SLE patients that circulating apoptotic MPs activate dendritic cell subsets and prime neutrophils [110]. Moreover, Winber et al. found that SLE patients display an increased production of ROS and degranulation by polymorphonuclear leukocytes (PMNs) in response to MPs [131]. Finally, Salvi et al. observed an increased secretion of IFN-α from blood-found plasmacytoid dendritic cells (pDCs) as a response to microRNAs carried by exosomes in SLE patients. They confirmed that exosome-delivered miRNAs could engage TLR7 endogenously to induce pDC activation in these patients [132]. A broad range of effects has brought about, for exosomes, an increasing interest, and curiosity of researchers for developing therapeutic strategies for various diseases such as cancer, cardiovascular diseases, nephropathies, and autoimmune diseases [133]. Exos could be an interesting target for treating autoimmune reactions in SLE patients.

## Double edge sword of IEXs therapy

Exosomes are released from all eukaryotic cells and carry different cargoes depending on their nature (e.g., transformed, differentiated, stimulated, and stressed). Since healthy cells produce 10^3^-10^4^ exosomes per cell, cancer or tumor cells produce more significant amounts in that tissue or another tissue [134]. Unlike normal cells-derived exosomes, which can be used in therapeutic fields, tumors and hyperactivated immune system cells-derived exosomes (IEXs) can steer the cells to exhibit inappropriate and dangerous effects [135, 136]. It is also proposed that IEXs may trigger undesired responses such as acceleration or prevention of some gene expression and cytokine regulation. IEXs have a wide range of activities, such as some gene expression immune system fluctuation, antitumor immunity modulation, and excluding the immune system, activation/inactivation of immune responses in antigen-presenting cells (APCs) [137]. Interestingly, while some IEXs suppress immune responses to raise homeostasis, others trigger physiologic and pathologic inflammatory responses (leading to removing pathogens or tissue destruction, autoimmunity, and allergies). For instance, immune cells infiltrating the tumor microenvironment actively communicate via exosomes to accelerate tumor progression and regulate the malignancy cascade by inducing cell proliferation/migration/invasion, angiogenesis, or metastasis [138, 139]. Therefore, exosomes can be regarded as a sign of an ignited cancer and specific other immune-related diseases such as asthma [140] and cardiovascular disease [135, 141]. Therefore, engineered IEXs loaded with special cargo are considered a replacement for improving their effects [142]. This problem also highlights the need to select an appropriate exosome source to maximize therapeutic potential. Finally, despite all the advantages of IEXs, there are still many uncertainties about the clinical applications of these vesicles and their effect on the affected people [143]. In this regard, choosing the appropriate origin for exosome isolation for a specific therapeutic application, as well as the right isolation method and preventing damage to it, is an issue that should be given a lot of attention.

## Conclusion and perspectives

Despite all efforts to develop ways to prevent, diagnose, and treat autoimmune diseases, we do not yet have a definite or accurate picture of these pathologies. Patients continue to suffer due to suboptimal treatment strategies. Exosomes have recently been identified as potential biomarkers, strong immune stimuli, drug carriers, and substantial role players in physiological and pathological processes. Through their unique properties, exosomes induce macrophage polarization and phenotypic changes, halt dendritic cells’ maturation and antigen presentation capacities, and suppress adaptive immunity by inhibiting T and B cell activation. These nanovesicles exert their immunoregulatory effects in many autoimmune diseases through their contained protein, DNA, and RNA (especially miRNA). The extensive clinical applications of exosomes as endogenous vesicles capable of carrying biopharmaceuticals have made them complementary or alternative therapies in autoimmune diseases. Specifically, mesenchymal stem cell (MSCs) derived exosomes show exquisite therapeutic potential in autoimmune diseases. Several unique exosome features, such as their abundance in various body fluids and their stability at -80 ° C, have made them a potent candidate for successful SLE treatment. They also have a long half-life in the body and can protect their internal materials and contents against enzymatic digestion. They can be readily modified based on the intended target cell profile.

Numerous challenges remain to be addressed before exosomes are successfully brought to the clinic. Most pressingly, more needs to be understood about the properties of exosomes from different cell sources and how this impacts target cell functions. Second, separation, quantification, and analysis methods of exosome contents need to be standardized within the field. Storage methods and delivery approaches must be considered as exosomes are brought to the clinic, which could have major impacts.

## Data Availability

All data generated or analyzed during this review are included in published articles.
